# Supplementary material for: Genome Characterization of Bird-Related Rhabdoviruses Circulating in Africa
Source: Viruses. 2021 Oct 27;13(11):2168. doi: 10.3390/v13112168 (PMC8622386; doi:10.3390/v13112168)

Figure S1. Reads coverage of the genome sequences of the seven bird-related sunrhaviruses. The coverage is presented after a last mapping step using initial NGS data.

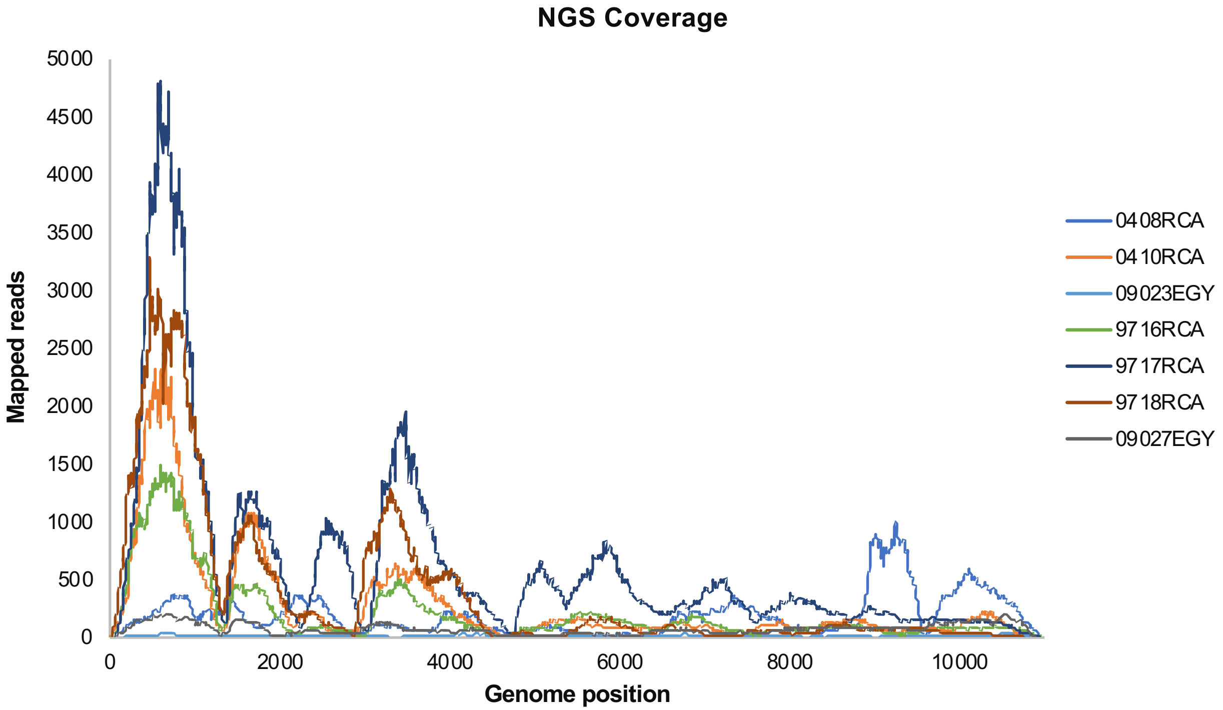

Supplement: Supplementary file 1 [file viruses-13-02168-s001.zip › Figure_S1_110921.pdf]
