# Supplementary material for: Genome Characterization of Bird-Related Rhabdoviruses Circulating in Africa
Source: Viruses. 2021 Oct 27;13(11):2168. doi: 10.3390/v13112168 (PMC8622386; doi:10.3390/v13112168)

Figure S2. Alignment and genetic comparison of the C proteins from sunrhaviruses and tupaviruses. Multiple amino acid sequences alignment of the C proteins from the 14 sunrhabivruses (including the 7 newly bird-related viruses) or the 3 tupaviruses (with DUV, KLAV and TUPV), performed using Clustal Omega (default parameters, SnapGene, v. 5.3.2) and three different datasets (a = 11 sequences with BBOV, BEAV, GARV, HDV, KOLV, MTYV, NASV, OUAV, SJAV, SUNV and WCV; b = 3 sequences with DDV, KWAV and OVRV; c = 3 sequences with DURV, KLAV and TUPV). Aligned sequences were edited using MView (https://www.ebi.ac.uk/Tools/msa/mview/), with the corresponding amino acid color code (bright green: hydrophobic, dark green: large hydrophobic, purple: polar, bright blue: negative charge, dull blue: small alcohol, red: positive charge, yellow: cystein).

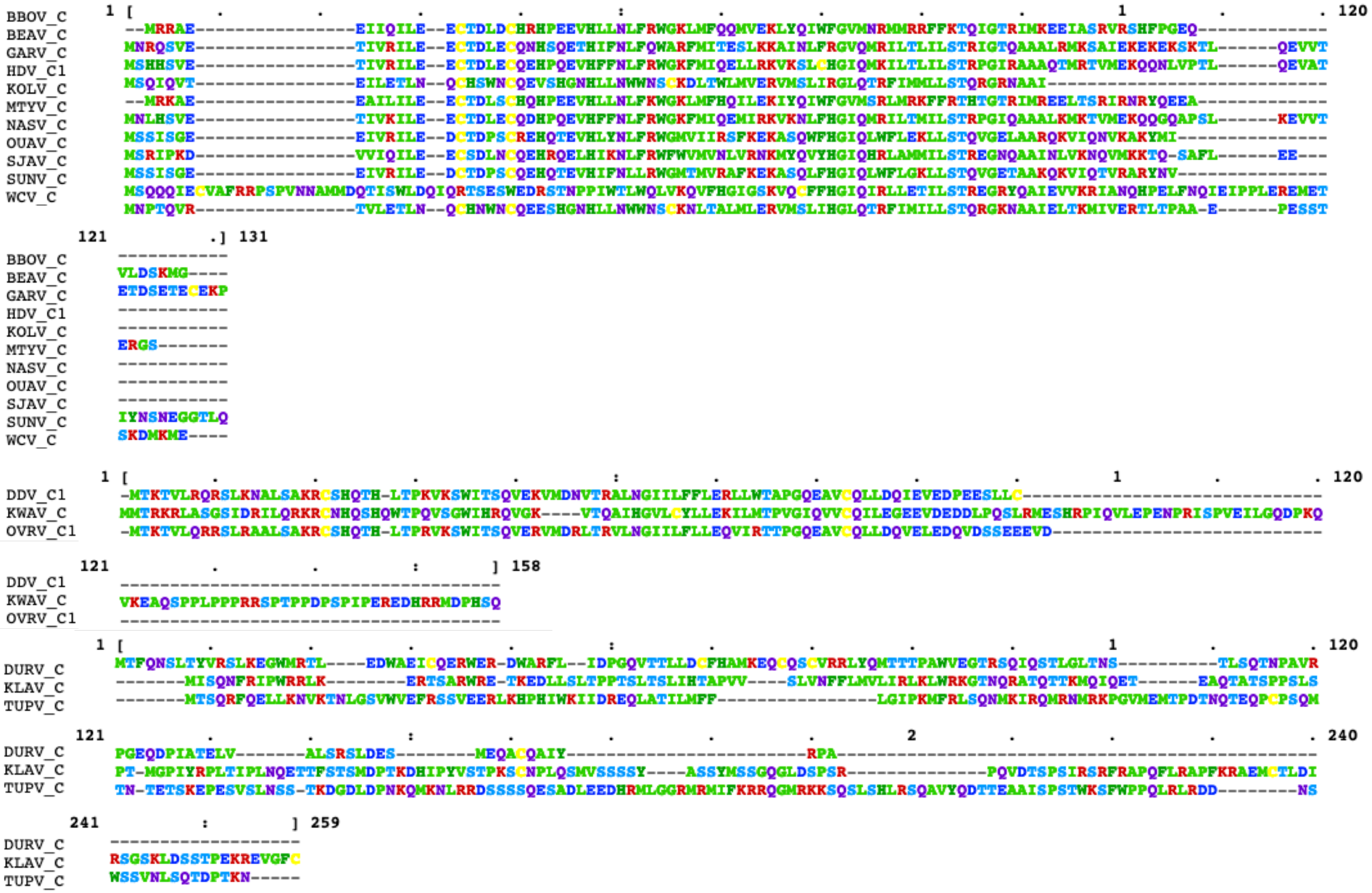

Supplement: Supplementary file 1 [file viruses-13-02168-s001.zip › Figure_S2_240921.pdf]
