# Supplementary material for: Genome Characterization of Bird-Related Rhabdoviruses Circulating in Africa
Source: Viruses. 2021 Oct 27;13(11):2168. doi: 10.3390/v13112168 (PMC8622386; doi:10.3390/v13112168)

Figure S3. Alignment and genetic comparison of the SH proteins from sunrhaviruses and tupaviruses. Multiple amino acid sequences alignment of the SH proteins from the 14 sunrhaviruses (including the 7 newly bird-related viruses) or the 3 tupaviruses (with DUV, KLAV and TUPV), performed using Clustal Omega (default parameters, SnapGene, v. 5.3.2). Due to the high genetic diversity, the 3 tupaviruses DUV, KLAV and TUPV compared to the 14 sunrhaviruses, they were aligned separately. Aligned sequences were edited using MView (https://www.ebi.ac.uk/Tools/msa/mview/), with the corresponding amino acid color code (bright green: hydrophobic, dark green: large hydrophobic, purple: polar, bright blue: negative charge, dull blue: small alcohol, red: positive charge, yellow: cystein).

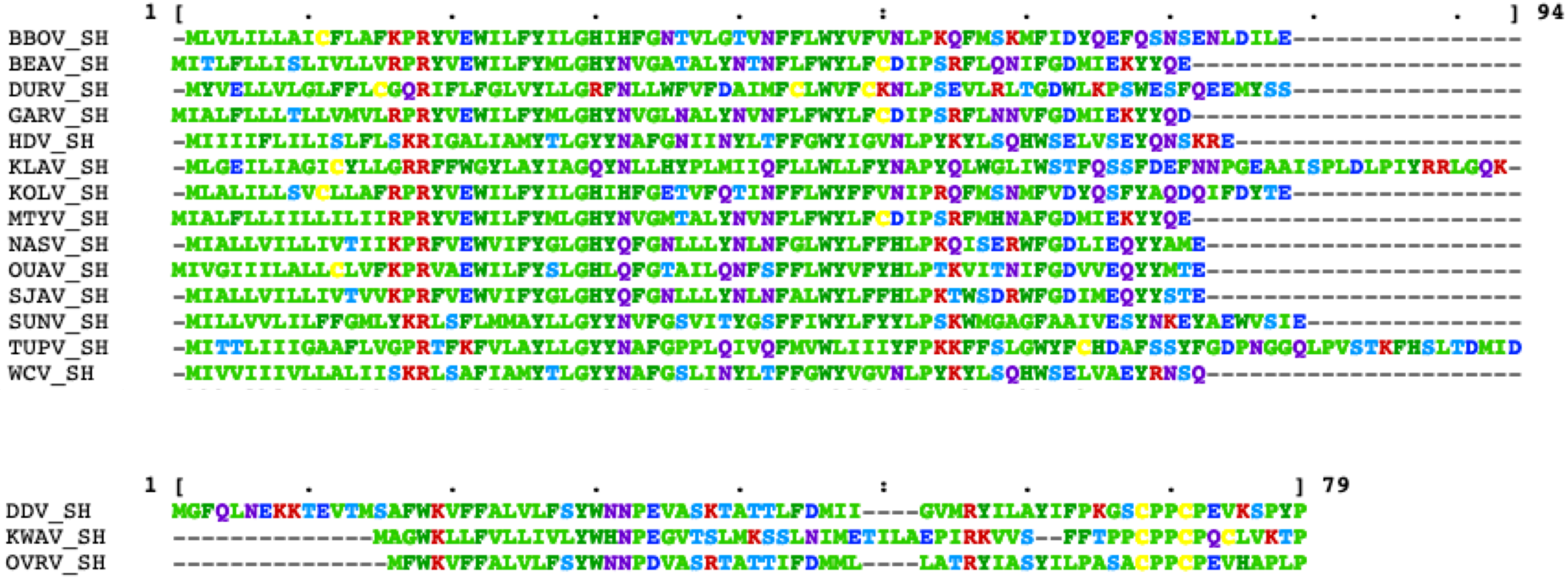

Supplement: Supplementary file 1 [file viruses-13-02168-s001.zip › Figure_S3_191021.pdf]
