# Supplementary material for: Genome Characterization of Bird-Related Rhabdoviruses Circulating in Africa
Source: Viruses. 2021 Oct 27;13(11):2168. doi: 10.3390/v13112168 (PMC8622386; doi:10.3390/v13112168)
Supplement: Supplementary file 1 [file viruses-13-02168-s001.zip › Figure_S4_240921.pdf]

Figure S4. Description of the transcription initiation (TI) and the transcription termination (TTP) signal sequences of the genes of the seven bird-related sunrhaviruses, and their positions related to the ATG of the associated ORF. Conserved motifs are indicated in grey for TI or underlined for TTP. The consensus sequences are indicated in italic.
[a] The beginning of the N gene is lacking in the genome sequence obtained by NGS analysis.
[b] The position of the TTP and the TI signals are inverted in the genome sequence (the TTP sequence of the SH (U3) gene being after the TI sequence of the G gene, with the sequence ATCA between them).
[c] Beginning of the genome sequence obtained from GenBank number MK965544.
[d] Presence of a putative additional ORF upstream the N gene.
D: A, G or T; W: A or T; Y: C or T; S: C, G or T; H: A, C or T; M: A or C.

SAJV_0408RCA

Leader – N[a]	
N - P	CATGAAAAAAACAACA - N_6 - ATG
P - M	CATGAAAAAAACAACA - N_9 - ATG
M – SH (U2)	CATGAAAAAAACAACA - N_9 - ATG
SH (U2) - G	CTTGAAAAAAACAACA - N_9 - ATG
G - L	CATGAAAAAAACAACA - N_9 - ATG
L - Trailer	CGCGAAAAAAA
Consensus	*CDTGAAAAAAACAACA*

BBOV_9716RCA

Leader – N	GAACA - N_6 - ATG
N – P	CATGAAAAAAA CAACA - N_9 - ATG
P – M	CATGAAAAAAA CAACA - N_9 - ATG
M – SH (U3)	CTTGAAAAAAA CAACA - N_9 - ATG
SH (U3) – G	CATGAAAAAAA CAACA - N_9 - ATG
G – L	CATGAAAAAAAACTAACA - N_9 - ATG
L –Trailer	CATGAAAAAAA
Consensus	*CWTGAAAAAAA* S*AACA*

NASV_0410RCA

Leader – N[a]	
N - P	CATGAAAAAAACAACA - N_6 - ATG
P - M	CATGAAAAAAACAACA - N_9 - ATG
M – SH (U2)	CATGAAAAAAACAACA - N_9 - ATG
SH (U2) - G	CATGAAAAAAACAACA - N_9 - ATG
G - L	CATGAAAAAAATAACA - N_9 - ATG
L - Trailer	CTTGAAAAAAA
Consensus	*CWTGAAAAAAAYAACA*

KOLV_9717RCA

Leader – N	CATGAAAAAAA CAACA - N_6 - ATG
N – P	CATGAAAAAAA CAACA - N_9 - ATG
P – M	CATGAAAAAAA CAACA - N_9 - ATG
M – SH (U2)	CTTGAAAAAA CAACA - N_9 - ATG
SH (U2) – G	CATGAAAAAAA CAACA - N_9 - ATG
G – L	CATGAAAAAAAACTAACA - N_9 - ATG
L –Trailer	CATGAAAAAAA
Consensus	*CWTGAAAAAAA YAACA*

OUAV_9718RCA

Leader – N	CAACA - N_3 - ATG
N – P	CATGAAAAAAATAACA - N_3 - ATG
P – M	TTTGAAAAAAACAACA - N_6 - ATG
M – SH (U3)	AATAAAAAAAACAACA - N_9 - ATG
SH (U3) – G[b]	CATGAAAAAAACAACA - N_3 - ATG
G – L	CATGAAAAAAACAACA - N_9 - ATG
L –Trailer	CTTGAAAAAAA
Consensus	*HWTGAAAAAAAYAACA*

BEAV_09023EGY

Leader – N[c]	AACA - N_6 - ATG
N – P	CATGAAAAAAACAACA - N_3 - ATG
P – M	CATGAAAAAAACAACA - N_9 - ATG
M – SH (U3)	CATGAAAAAAACAACA - N_6 - ATG
SH (U3) – G	CTTGAAAAAAACAACA - N_9 - ATG
G – L	CATGAAAAAAACAACA - N_9 - ATG
L –Trailer	CATGAAAAAAAA
Consensus	*CWTGAAAAAAAMAACA*

MTYV_09027EGY

Leader – N	GAACA - N_6 - ATG
N – P	CATGAAAAAAACAACA - N_3 - ATG
P – M	CATGAAAAAAACAACA - N_9 - ATG
M – SH (U2)	ATTGAAAAAAACAACA - N_6 - ATG
SH (U2) – G	CTTGAAAAAAACAACA - N_9 - ATG
G – L	CATGAAAAAAACAACA - N_9 - ATG
L –Trailer	CATGAAAAAAA
Consensus	*MWTGAAAAAAASAACA*
