# Supplementary material for: Genome Characterization of Bird-Related Rhabdoviruses Circulating in Africa
Source: Viruses. 2021 Oct 27;13(11):2168. doi: 10.3390/v13112168 (PMC8622386; doi:10.3390/v13112168)
Supplement: Supplementary file 1 [file viruses-13-02168-s001.zip › Table_S1_220921.pdf]

**Table S1.** Description of the primers specifically designed to fulfil gaps into the genome sequences generated after NGS sequencing.

| Virus | Primer | Sequence | Size (nt) | Position |
|---|---|---|---|---|
| BBOV_9716RCA | 9716_GSP1_smart | 5' GGATCGGCATTTTGTGAAGAGGTAGAGG 3' | 384 | 1 nt – 384 nt (N gene) |
| | 9716_NGSP1_smart | 5' CATTCCTTGAACCGATCCTCCGACC 3' | | |
| OUAV_9718RCA | 9718_GSP1_smart | 5' CAGCCCGCAACAGAACACATACATCC 3' | 461 | 1 nt – 461 nt (N gene) |
| | 9718_NGSP1_smart | 5' GGATTTATGTCCTCACCTTGCCTTCC 3' | | |
| | 9718-F1 | 5' GATCACATTTCATTGGCAATGC 3' | 347 | 4287 nt – 4633 nt (G gene) |
| | 9718-R1 | 5' GAGTCTTGTTGGTTTCTCTGAAGATG 3' | | |
| | 9718-F2 | 5' GGATTGATTACCACAAGTTAG 3' | | |
| | 9718-R2 | 5' CAAGTTCAGATATTCTGTATTCC 3' | | |
| MTYV_09027EGY | MTYV_GSP1_smart | 5' CAATCCGATGCAATAAACAAACATCCAC 3' | 459 | 1 nt – 459 nt (N gene) |
| | MTYV_NGSP1_smart | 5' CCTCTATTTCACAAAGATTCCAAGGGGC 3' | | |
