# Supplementary material for: Genome Characterization of Bird-Related Rhabdoviruses Circulating in Africa
Source: Viruses. 2021 Oct 27;13(11):2168. doi: 10.3390/v13112168 (PMC8622386; doi:10.3390/v13112168)
Supplement: Supplementary file 1 [file viruses-13-02168-s001.zip › Table_S4_220921.pdf]

Table S4. Description of the C proteins from sunrhaviruses and tupaviruses. The predicted molecular weights (MW), isoelectric points (pI) and charge values (at pH 7) were determined using SnapGene software (version 5.2.3). The calculation of the instability, aliphatic and grand average of hydropathicity (GRAVY) was performed using the ProtParam tool on the ExPASy server (https://www.expasy.org/resources/protparam). The identification of transmembrane helices was done with the TMHMM Server (version 2.0) (http://www.cbs.dtu.dk/services/TMHMM-2.0/) and the TMpred server on the ExPASy server (https://embnet.vital-it.ch/software/TMPRED_form.html). The putative protein topology was obtained using the normal prediction of the Phoebius server (https://phobius.sbc.su.se/index.html).

Virus	Genus	GenBank number	ORF length (nt)	Protein length (aa)	Protein MW (kDa)	pI	Charge (pH 7)	Instability index	Aliphatic index	GRAVY Index	TMHMM helix nb. (location)	Tmpred helix nb.	Phoebius prediction, aa position (aa number)			
													Signal peptide	Non cyto.	Trans. helix	Cyto.
BBOV	*Sunrhavirus*	MW491756	264	87	10.7	7.7	1.76	Unstable	78.39	-0.476	0 (non cyto.)	0		1-87 (87)		
BEAV	*Sunrhavirus*	MW491759	312	103	11.9	8.71	2.19	Unstable	98.45	-0.190	0 (non cyto.)	0		1-103 (103)		
DDV	*Sunrhavirus*	MG251664	267	88	10	6.86	-0.43	Stable	104.09	-0.150	0 (cyto.)	0				1-88 (88)
DURV	*Tupavirus*	FJ952155	411	136	15.6	4.69	-6.93	Unstable	71.76	-0.476	0 (non cyto.)	0		1-136 (136)		
GARV	*Sunrhavirus*	NC_055530	324	107	12.4	6.17	-4.18	Unstable	88.32	-0.307	0 (non cyto.)	0		1-107 (107)		
HDV	*Sunrhavirus*	KJ432573	309	74	8.7	6.96	-0.15	Stable	97.43	-0.107	0 (non cyto.)	0		1-74 (74)		
KLAV	*Tupavirus*	NC_034549	645	214	24.1	10.53	10.58	Unstable	67.90	-0.512	1	1		1-39 (39)	40-60 (21)	61-214 (154)
KOLV	*Sunrhavirus*	MW491757	225	87	10.7	7.75	2.05	Unstable	86.32	-0.463	0 (non cyto.)	0		1-87 (87)		
KWAV	*Sunrhavirus*	KM204985	465	154	17.6	7	0.01	Unstable	80.91	-0.788	0 (non cyto.)	0				1-154 (154)
MTYV	*Sunrhavirus*	MW491760	303	100	11.6	7.7	1.76	Unstable	90.60	-0.195	0 (non cyto.)	0		1-100 (100)		
NASV	*Sunrhavirus*	MW491755	264	87	10.2	7.18	0.47	Unstable	96.32	-0.161	0 (non cyto.)	0		1-87 (87)		
OUAV	*Sunrhavirus*	MW491758	279	92	11	8.71	2.76	Unstable	97.39	-0.297	0 (non cyto.)	0		1-92 (92)		
OVRV	*Sunrhavirus*	NC_025399	276	91	10.4	6.24	-1.81	Unstable	104.84	-0.300	0 (cyto.)	0				1-91 (91)
SJAV	*Sunrhavirus*	MW491754	264	87	10	7.18	0.47	Unstable	91.84	-0.145	0 (non cyto.)	0		1-87 (87)		
SUNV	*Sunrhavirus*	NC_025401	396	131	15.3	5.65	-3.52	Unstable	84.05	-0.492	0 (non cyto.)	0		1-131 (131)		
TUPV	*Tupavirus*	NC_007020	666	221	25.8	10.31	9.38	Unstable	58.64	-0.968	0 (non cyto.)[a]	1		1-44 (44)	45-62 (18)	63-221 (159)
WCV	*Sunrhavirus*	NC_028232	309	102	11.7	6.79	-0.85	Unstable	85.10	-0.380	0 (non cyto.)	0		1-102 (102)		

a: Detection of two transmembrane helices in total but without significative probability.
