# Supplementary material for: Genome Characterization of Bird-Related Rhabdoviruses Circulating in Africa
Source: Viruses. 2021 Oct 27;13(11):2168. doi: 10.3390/v13112168 (PMC8622386; doi:10.3390/v13112168)
Supplement: Supplementary file 1 [file viruses-13-02168-s001.zip › Table_S5_220921.pdf]

Table S5. Description of the SH proteins from sunrhaviruses and tupaviruses. The predicted molecular weights (MW), isoelectric points (pI) and charge values (at pH 7) were determined using SnapGene software (version 5.2.3). The calculation of the instability, aliphatic and grand average of hydropathicity (GRAVY) was performed using the ProtParam tool on the ExPASy server (https://www.expasy.org/resources/protparam). The identification of transmembrane helices was done with the TMHMM Server (version 2.0) (http://www.cbs.dtu.dk/services/TMHMM-2.0/) and the TMpred server on the ExPASy server (https://embnet.vital-it.ch/software/TMPRED_form.html). The putative protein topology was obtained using the normal prediction of the Phoebius server (https://phobius.sbc.su.se/index.html).

Virus	Genus	GenBank number	ORF length (nt)	Protein length (aa)	Protein MW (kDa)	pI	Charge (pH 7)	Instability index	Aliphatic index	GRAVY Index	TMHMM helix nb.	Tmpred helix nb.	Phoebius prediction, aa position (aa number)			
													Signal peptide	Non cyto.	Trans. helix	Cyto.
BBOV	*Sunrhavirus*	MW491756	234	77	9.2	5.52	-2,21	Unstable	121.43	0.718	2	2	1-13 (13)	14-22 (9)	23-49 (27)	50-77 (28)
BEAV	*Sunrhavirus*	MW491759	216	71	8.6	5.56	-1.49	Unstable	123.52	0.665	0[a]	2	1-20 (20)	21-29 (9)	30-49 (20)	50-71 (22)
DDV	*Sunrhavirus*	MG251664	228	75	8.6	7.45	0.61	Unstable	76.67	0.228	0[a]	2		31-41 (11)	12-30 (19)<br>42-59 (18)	1-11 (11)<br>60-75 (16)
DURV	*Tupavirus*	FJ952155	234	77	9.2	4.47	-4	Unstable	115.06	0.790	2	1		27-31 (5)	5-26 (22)<br>32-48 (17)	1-4 (4)<br>49-77 (29)
GARV	*Sunrhavirus*	NC_055530	216	71	8.7	5.51	-1.49	Unstable	126.20	0.708	1[a]	2	1-20 (20)	21-29 (9)	30-49 (20)	50-71 (22)
HDV	*Sunrhavirus*	KJ432573	222	73	8.6	9.36	2.12	Unstable	124.25	0.523	2	2	1-19 (19)	20-28 (9)	29-49 (21)	50-73 (24)
KLAV	*Tupavirus*	NC_034549	279	92	10.8	6.64	-0.49	Unstable	116.74	0.420	2	2		1-5 (5)<br>52-92 (41)	6-25 (20)<br>32-51 (20)	26-31 (6)
KOLV	*Sunrhavirus*	MW491757	234	77	9.5	4.65	-3.21	Unstable	103.77	0.609	1	2	1-19 (19)	20-28 (9)	29-49 (21)	50-77 (28)
KWAV	*Sunrhavirus*	KM204985	192	63	7.1	7.12	0.28	Unstable	111.27	0.565	0	1	1-24 (24)	25-63 (39)		
MTYV	*Sunrhavirus*	MW491760	216	71	8.7	6.35	-1.21	Unstable	126.34	0.793	2	2	1-16 (16)	17-31 (15)	32-49 (18)	50-71 (22)
NASV	*Sunrhavirus*	MW491755	216	71	8.6	6.55	-0.59	Unstable	134.51	0.708	2	2	1-19 (19)	20-28 (9)	29-49 (21)	50-71 (22)
OUAV	*Sunrhavirus*	MW491758	219	72	8.5	6.35	-1.21	Unstable	129.86	0.969	2	2	1-20 (20)	21-29 (9)	30-50 (21)	51-72 (22)
OVRV	*Sunrhavirus*	NC_025399	183	60	6.8	6.4	-1.11	Unstable	91.17	0.595	0	2	1-23 (23)	24-60 (37)		
SJAV	*Sunrhavirus*	MW491754	216	71	8.6	6.55	-0.59	Stable	120.70	0.594	2	2	1-16 (16)	17-31 (15)	32-49 (18)	50-71 (22)
SUNV	*Sunrhavirus*	NC_025401	237	78	9.3	6.26	-0.17	Unstable	113.72	0.929	2	2	1-19 (19)	20-28 (9)	29-51 (23)	52-78 (27)
TUPV	*Tupavirus*	NC_007020	282	93	10.6	7.49	0.79	Stable	100.65	0.671	2	2	1-20 (20)	21-29 (9)	30-51 (22)	52-93 (42)
WCV	*Sunrhavirus*	NC_028232	216	71	8.3	9.39	2.12	Stable	130.42	0.755	2	2	1-19 (19)	20-28 (9)	29-49 (21)	50-71 (22)

a: Detection of two transmembrane helices in total but without significative probability.
